# Effect of Warfarin Treatment on Survival of Patients With Pulmonary Arterial Hypertension (PAH) in the Registry to Evaluate Early and Long-Term PAH Disease Management (REVEAL)

**DOI:** 10.1161/CIRCULATIONAHA.115.018435

**Published:** 2015-12-21

**Authors:** Ioana R. Preston, Kari E. Roberts, Dave P. Miller, Ginny P. Sen, Mona Selej, Wade W. Benton, Nicholas S. Hill, Harrison W. Farber

**Affiliations:** From Tufts University School of Medicine, Boston, MA (I.R.P., K.E.R., N.S.H.); ICON Clinical Research, San Francisco, CA (D.P.M., G.P.S.); Actelion Pharmaceuticals US Inc., South San Francisco, CA (M.S., W.W.B.); and Boston University School of Medicine, Boston, MA (H.W.F.).

**Keywords:** anticoagulants, hypertension, pulmonary, survival, therapy

## Abstract

Long-term anticoagulation is recommended in idiopathic pulmonary arterial hypertension (IPAH). In contrast, limited data support anticoagulation in pulmonary arterial hypertension (PAH) associated with systemic sclerosis (SSc-PAH). We assessed the effect of warfarin anticoagulation on survival in IPAH and SSc-PAH patients enrolled in Registry to Evaluate Early and Long-term PAH Disease Management (REVEAL), a longitudinal registry of group I PAH.

**Methods and Results—:**

Patients who initiated warfarin on study (n=187) were matched 1:1 with patients never on warfarin, by enrollment site, etiology, and diagnosis status. Descriptive analyses were conducted to compare warfarin users and nonusers by etiology. Survival analyses with and without risk adjustment were performed from the time of warfarin initiation or a corresponding quarterly update in matched pairs to avoid immortal time bias. Time-varying covariate models were used as sensitivity analyses. Mean warfarin treatment was 1 year; mean international normalized ratios were 1.9 (IPAH) and 2.0 (SSc-PAH). Two-thirds of patients initiating warfarin discontinued treatment before the last study assessment. There was no survival difference with warfarin in IPAH patients (adjusted hazard ratio, 1.37; *P*=0.21) or in SSc-PAH patients (adjusted hazard ratio, 1.60; *P*=0.15) in comparison with matched controls. However, SSc-PAH patients receiving warfarin within the previous year (hazard ratio, 1.57; *P*=0.031) or any time postbaseline (hazard ratio, 1.49; *P*=0.046) had increased mortality in comparison with warfarin-naïve patients.

**Conclusions—:**

No significant survival advantage was observed in IPAH patients who started warfarin. In SSc-PAH patients, long-term warfarin was associated with poorer survival than in patients not receiving warfarin, even after adjusting for confounders.

**Clinical Trial Registration—:**

URL: http://www.clinicaltrials.gov. Unique identifier: NCT00370214.

## Abstract 2

Supplemental Digital Content is available in the text.

Pulmonary arterial hypertension (PAH) is a fatal disease characterized by a vasoconstrictive, proliferative, thrombotic phenotype, leading to increased pulmonary artery pressure and pulmonary vascular resistance, and eventually to right ventricular failure.^1^ In addition to pulmonary vascular remodeling, the pathophysiology of PAH includes in situ thrombosis of the small pulmonary arteries.^2–4^ The World Symposium on Pulmonary Hypertension group I category includes several types of PAH with similar pathological changes.^5–7^ It is unclear whether thrombotic vascular lesions are an integral part of the pulmonary vascular pathology or are simply an epiphenomenon. In support of the former hypothesis are abnormalities of blood coagulation factors, antithrombotic factors, and the fibrinolytic system observed in patients with idiopathic PAH (IPAH).^8^ However, these abnormalities have not been described in other forms of group I PAH, including PAH associated with systemic sclerosis (SSc-PAH).

**Editorial see p 2360**

**Clinical Perspective on p 2411**

Based on these pathological considerations suggesting a prothrombotic milieu of the diseased pulmonary vasculature, 8 observational studies (2 prospective and 6 retrospective) have evaluated the effect of chronic anticoagulation on outcome in patients with IPAH; 6 of these studies reported positive outcomes.^9–16^ Most of these observational studies were published more than a decade ago, before the introduction of many current PAH therapies. However, despite the limitations of the available evidence, current treatment algorithms recommend warfarin in patients with IPAH.^17^ Data regarding anticoagulation in patients with associated forms of PAH such as SSc-PAH are even scarcer; however, such a strategy has been extrapolated from IPAH studies and current treatment algorithms recommend warfarin in these patients, based mostly on consensus opinion.^17^ Expert opinions on the benefit of warfarin in SSc-PAH and IPAH patients vary widely in regard to the effectiveness of treatment.^18^

Recently, the retrospective analysis from the European database Comparative, Prospective Registry of Newly Initiated Therapies for Pulmonary Hypertension (COMPERA) examined survival in patients with IPAH and SSc-PAH who received anticoagulation in comparison with patients who did not receive anticoagulation.^15^ In this analysis, survival improved in IPAH patients who received anticoagulation; however, anticoagulation was not associated with a survival benefit in patients with SSc-PAH.^15^ These observations are of particular concern because chronic anticoagulation can be associated with major fatal bleeding.^19^ Specifically, SSc-PAH patients with arteriovenous malformations of the gastrointestinal tract are at increased risk of serious bleeding.^20^ Last, all but 1 of the aforementioned studies were conducted before the availability of PAH-specific therapies,^7^ some of which have an antiplatelet effect and may alter the thrombotic profile of the pulmonary blood and vasculature. Therefore, in an era when multiple effective PAH therapies are available, the role of anticoagulation in the treatment of PAH is even more uncertain.

In an effort to reconcile the variable results and the limitations of previous studies, we used the large, multicenter, observational, US-based, longitudinal registry of patients with group I PAH, the Registry to Evaluate Early and Long-term PAH Disease Management (REVEAL Registry), to assess the association between chronic anticoagulation use with warfarin and outcomes in contemporary patients with IPAH and SSc-PAH.

## Methods

### REVEAL Registry

The design and baseline characteristics of patients enrolled in the REVEAL Registry have been described previously.^21^ In brief, the REVEAL Registry is a multicenter (55 sites, university-affiliated and community hospitals), observational, US-based study designed to provide information about demographics, disease course, and management of 3515 consecutively enrolled patients with newly or previously diagnosed group I PAH. The study was conducted in accordance with the amended Declaration of Helsinki and the protocol was reviewed by the institutional review board of each participating center with written informed consent obtained from all patients.^21^ Patients were followed for at least 5 years from the time of enrollment. Diagnosis was confirmed by right heart catheterization within 3 months before enrollment for newly diagnosed and >3 months before enrollment for previously diagnosed patients. PAH was defined as a mean pulmonary artery pressure >25 mm Hg at rest or >30 mm Hg with exercise, pulmonary capillary wedge pressure or left ventricular end-diastolic pressure ≤18 mm Hg, and pulmonary vascular resistance ≥240 dyn·sec·cm^–5^. The IPAH group included only patients with IPAH and did not include patients with heritable or anorexigen-induced PAH. SSc-PAH included patients with both limited and diffuse SSc-PAH.

Data were collected at the time of enrollment, followed by quarterly updates including information from any clinic visits or other patient contact in the previous 90 days. Exact dates of PAH-specific medications were collected throughout the study, whereas concomitant medications such as warfarin were identified at quarterly updates.

### Patients Who Initiated Warfarin On-Study and Patients Never on Warfarin

The patient cohort for this analysis was enrolled from 2006 to 2009. We identified 4 groups based on etiologic cohort and warfarin initiation. A nested design was used to match patients who initiated warfarin after REVEAL Registry enrollment to patients never on warfarin within each etiologic cohort (IPAH and SSc-PAH). These new warfarin users and patients never on warfarin were matched by a 1:1 ratio with the same enrollment center and exact etiology. Patients were also matched by diagnosis status (newly or previously diagnosed) at study enrollment and were followed on a quarterly basis. To adjust for warfarin discontinuation, a separate analogous time-varying covariate Cox proportional hazards model including all warfarin data within each etiologic cohort was performed as a sensitivity analysis. This model accounts for warfarin starts and stops at each quarter of data collection and includes all warfarin data until the last available quarter. To avoid immortal time bias,^22^ the time at risk was defined as the initiation of warfarin or at the matching quarterly update so that all survival follow-up was prospective (online-only Data Supplement Figure I). Thus, patients were excluded if they were already on warfarin at enrollment in both the matching-designed analysis and the sensitivity analysis.

### Statistical Analysis

All statistical comparisons were made between patients receiving and not receiving warfarin within etiology. Categorical data were presented as percentages and were analyzed using the χ^2^ or Fisher exact test where appropriate. Continuous data are summarized as mean±standard deviation or percentiles and were analyzed using the Student *t* test or Wilcoxon 2-sample rank sum test. Kaplan-Meier curves and estimates of survival were presented from the initiation of warfarin or the matching quarter to 36 months. Matching does not address potential confounding associated with differences in PAH severity. Therefore, univariable comparisons based on Kaplan-Meier curves and the log-rank test were supplemented with multivariable Cox proportional hazards models adjusting for differences in the risk profile. Risk adjustment to account for selection bias^23^ was conducted based on the REVEAL Registry prognostic equation^24^ at the quarterly update corresponding to warfarin initiation and match.

In addition to the main analysis, a multivariable Cox proportional hazards model including all warfarin data within each etiologic cohort was performed as a sensitivity analysis within both the IPAH and SSc-PAH subgroups. Warfarin use was modeled as time-interval time-varying covariates. In 2 different models, (1) past use (any on-study warfarin use) or (2) current use (any on-study warfarin use within the previous year) was compared with all patients who had no on-study warfarin use or patients who had no on-study warfarin use within the past year, respectively, as the main predictors in each model. PAH severity at baseline (study enrollment) was also accounted for after adjusting the models for diagnosis status, PAH medications, and risk profile at baseline. Survival was based on all-cause mortality, with patients censored only for loss to follow-up or at the close of study in December 2012. A *P* value of <0.05 was considered statistically significant.

## Results

### Patient Characteristics

**Figure 1.**
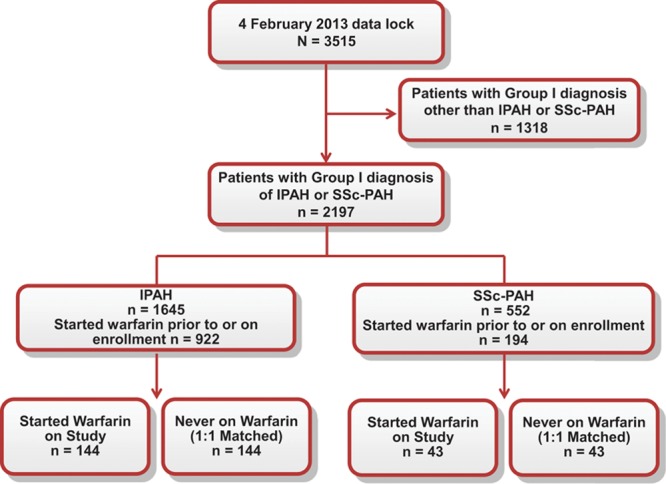
Study design. IPAH indicates idiopathic pulmonary arterial hypertension; and SSc-PAH, pulmonary arterial hypertension associated with systemic sclerosis.

A total of 2197 IPAH and SSc-PAH patients were enrolled in the REVEAL Registry: 1645 IPAH patients and 552 SSc-PAH patients. In this registry, 922 IPAH patients and 194 SSc-PAH patients started warfarin before or at enrollment. Of these, 163 IPAH patients and 55 SSc-PAH patients started warfarin after enrollment (postbaseline) and were included in the sensitivity analysis (Figure 1). Based on the matching criteria, we identified 144 IPAH and 43 SSc-PAH patients who initiated warfarin after enrollment (Figure 1) and were matched to 144 IPAH and 43 SSc-PAH patients, respectively, who were never on warfarin. These patients were included in the main analysis. Most patients were previously diagnosed with PAH at the time of enrollment into the REVEAL Registry. Table 1 describes the demographics and clinical characteristics of the 4 groups of PAH patients. With the exception of New York Heart Association (NYHA)/World Health Organization (WHO) functional class III/IV status and brain natriuretic peptide levels in the warfarin groups, characteristics were similar between the 2 pairs of groups. Notably, the REVEAL Risk Score was higher in the SSc-PAH warfarin cohort. Overall, the REVEAL Registry cohort was 72.4% white and 77.6% female. In the current analysis, no sex- or ethnicity-specific effects were observed. However, because of the small representation of some subgroups in this study, it is difficult to interpret whether these effects are truly absent.

**Table 1.**
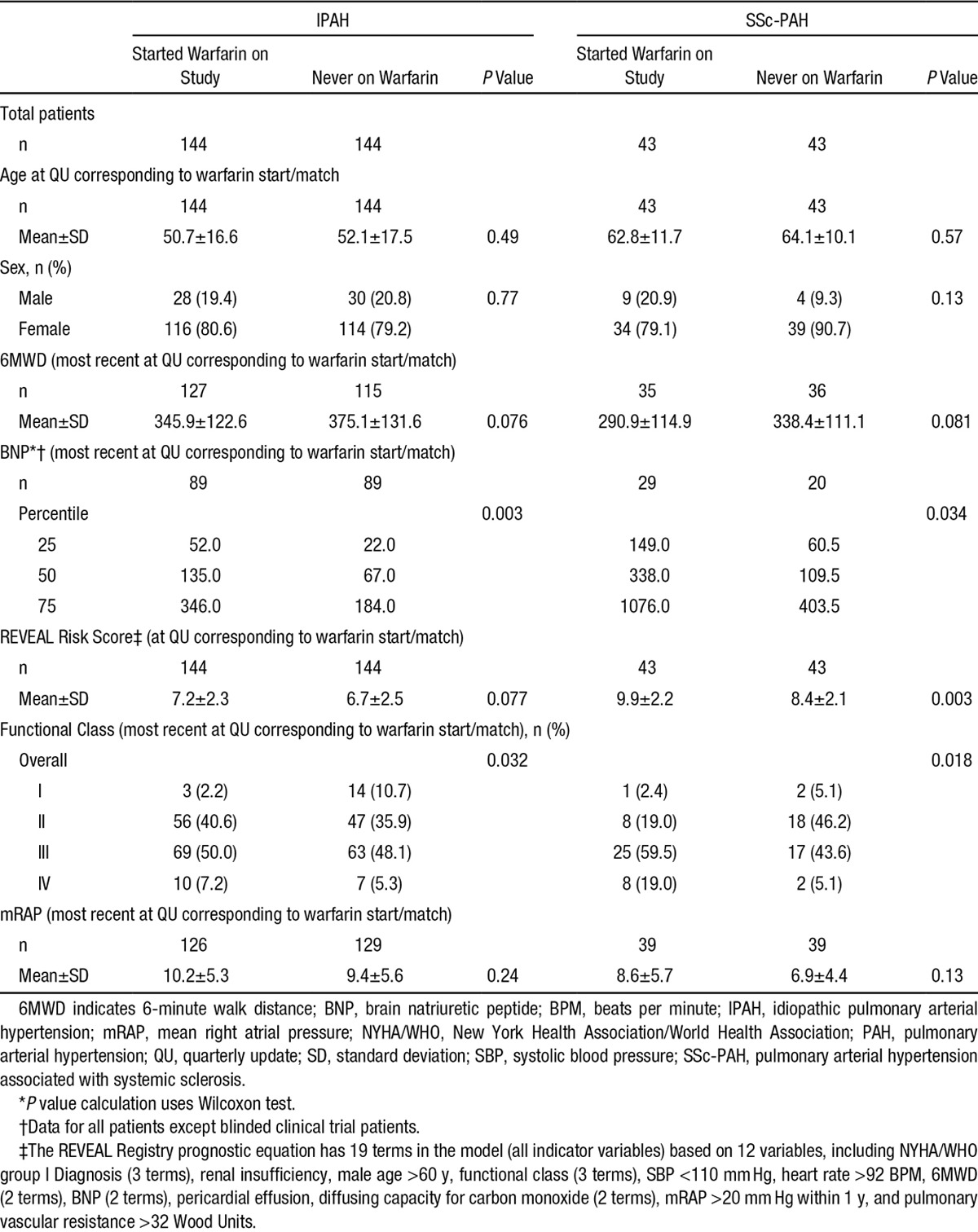
Patient Characteristics

Comorbid conditions were similar between the 2 sets of groups, including a low prevalence of thromboembolism (Table 2). PAH-specific medications are listed in Table 3. More patients in the warfarin groups received parenteral prostacyclins; (46.4% versus 15.1% for IPAH patients who started warfarin and never on warfarin, respectively, and 34.1% versus 14.6% SSc-PAH patients who started warfarin and never on warfarin, respectively). Moreover, patients in the warfarin groups received combination PAH-specific therapies more frequently (50.0% versus 40.2% for IPAH patients who started warfarin and never on warfarin, respectively, and 55.9% versus 25.6% SSc-PAH patients who started warfarin and never on warfarin, respectively). The mean international normalized ratio (INR) was 1.9 for IPAH and 2.0 for SSc-PAH patients (Table 4). In both IPAH and SSc-PAH warfarin-treated groups, the mean time on warfarin was 1 year. Time on warfarin ranged from 3 months to 42 months. Nearly two-thirds of patients discontinued warfarin before the last assessment (65.3% and 62.8% for IPAH and SSc-PAH, respectively) (Table 4).

**Table 2.**
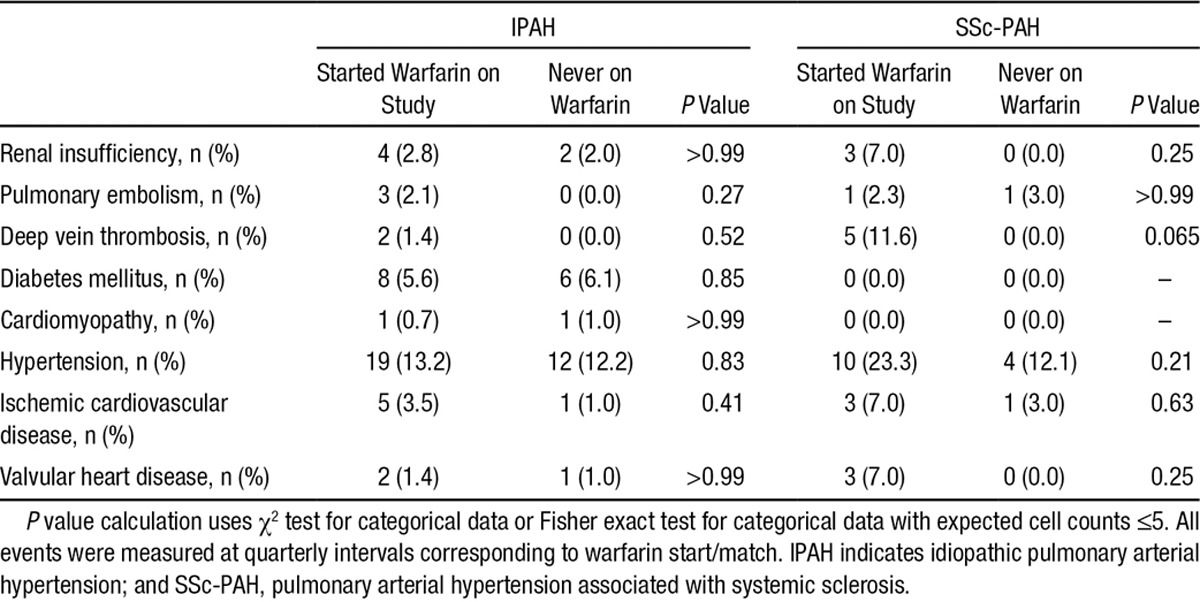
Patient Comorbidities

**Table 3.**
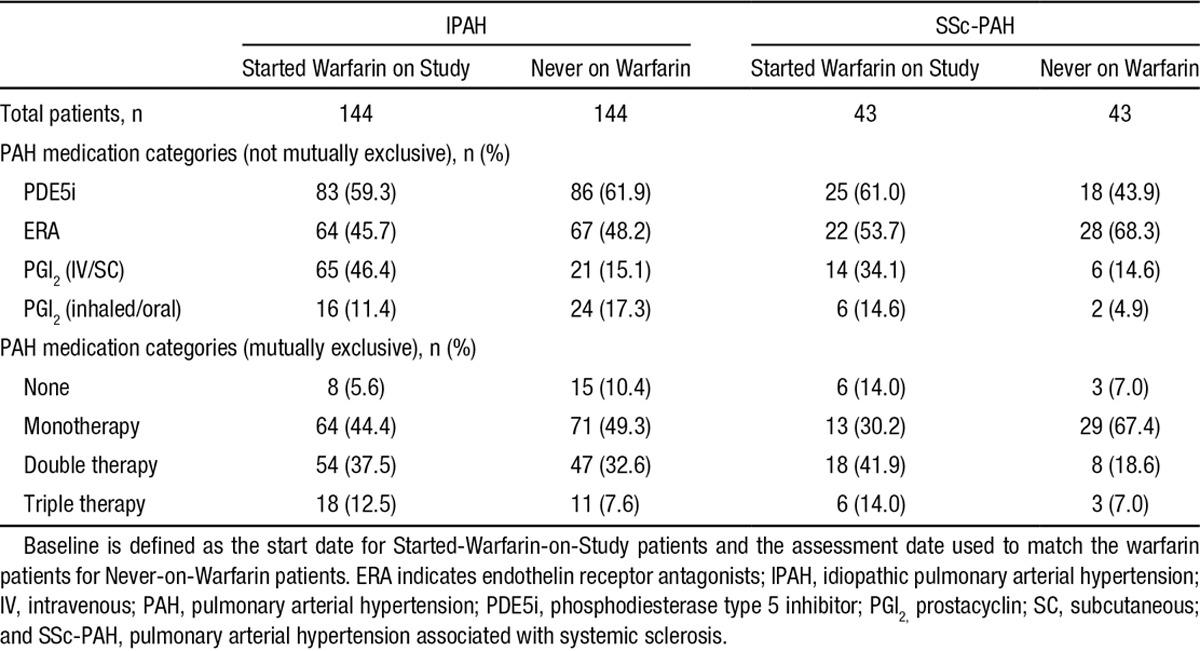
PAH Medications at Baseline

**Table 4.**
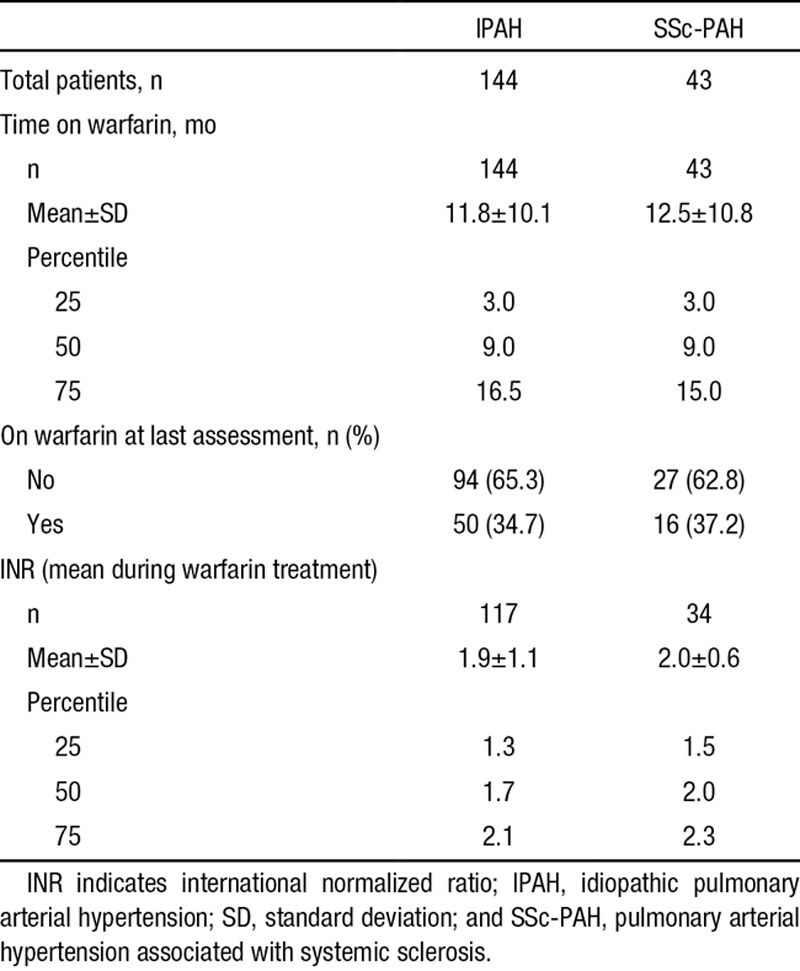
Warfarin Use

### Survival Analysis

**Figure 2.**
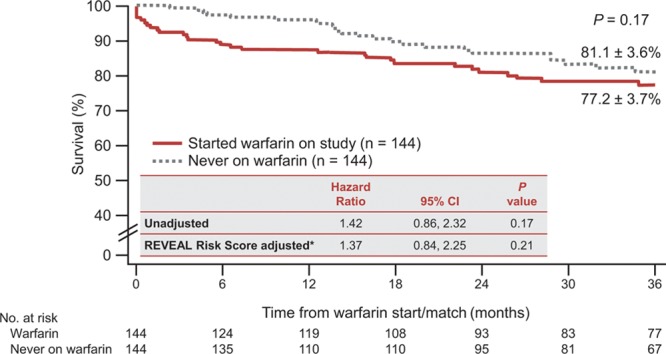
Kaplan-Meier estimates of survival at 36 months for IPAH patients. CI indicates confidence interval; and IPAH, idiopathic pulmonary arterial hypertension. *IPAH risk score at quarterly update corresponding to warfarin start.

**Figure 3.**
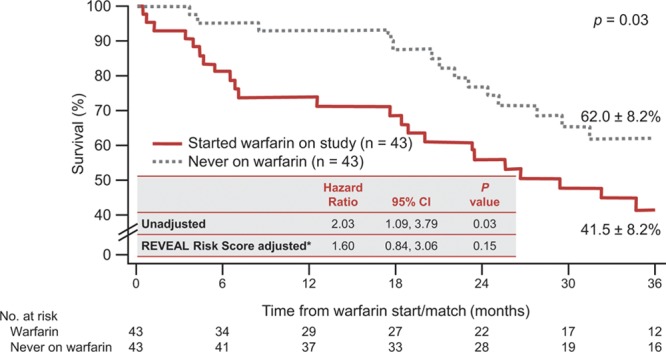
Kaplan-Meier estimates of survival at 36 months for SSc-PAH patients. CI indicates confidence interval; and SSc-PAH, pulmonary arterial hypertension associated with systemic sclerosis. *IPAH risk score at quarterly update corresponding to warfarin start.

Survival was assessed over the duration of the study. In IPAH patients, warfarin treatment was not significantly associated with survival at the end of the study in either the unadjusted Cox proportional hazards model (hazard ratio [HR], 1.42; 95% confidence interval [CI], 0.86–2.32; *P*=0.17) or the adjusted (HR, 1.37; 95% CI, 0.84–2.25; *P*=0.21) analysis for risk factors (Figure 2). In SSc-PAH patients, the unadjusted survival analysis showed that warfarin use was associated with significantly lower survival compared with matched controls (HR, 2.03; 95% CI, 1.09–3.79; *P*=0.03). However, when adjusted for the REVEAL Risk Score, there was no statistical difference between the 2 groups (HR, 1.60; 95% CI, 0.84–3.06; *P*=0.15) (Figure 3).

Matching analysis design results in the loss of information for unmatched patients and does not account for the time of warfarin discontinuation. Because there was a high warfarin discontinuation rate in the REVEAL Registry, a time-varying covariate model of the longitudinal effect of warfarin use was used to overcome this limitation. This model accounts for warfarin starts and stops and was used as a sensitivity analysis (online-only Data Supplement Table I). After adjusting for diagnosis status, PAH medication, and REVEAL Risk Score in the sensitivity analysis, the model affirmed the results in the main analysis in the IPAH cohort: there was no survival advantage in IPAH patients who were current (HR, 0.96; 95% CI 0.66–1.39, *P*=0.82) or past warfarin users (HR, 0.84; 95% CI, 0.59–1.20, *P*=0.34) in comparison with IPAH patients who had no on-study warfarin use within the past year or who had no on-study warfarin use, respectively (online-only Data Supplement Table I). On the contrary, SSc-PAH patients receiving warfarin within the previous year (HR, 1.57; 95% CI, 1.04–2.36; *P*=0.031) or at any time postbaseline (HR, 1.49; 95% CI, 1.01–2.20; *P*=0.046) had a poorer survival outcome than patients who had no on-study warfarin use within the past year or who had no on-study warfarin use, respectively, even after adjusting for diagnosis status, PAH medication, and REVEAL Risk Score (online-only Data Supplement Table I).

Causes of death are presented in Table 5. The majority of deaths were attributed to worsening of PAH.

**Table 5.**
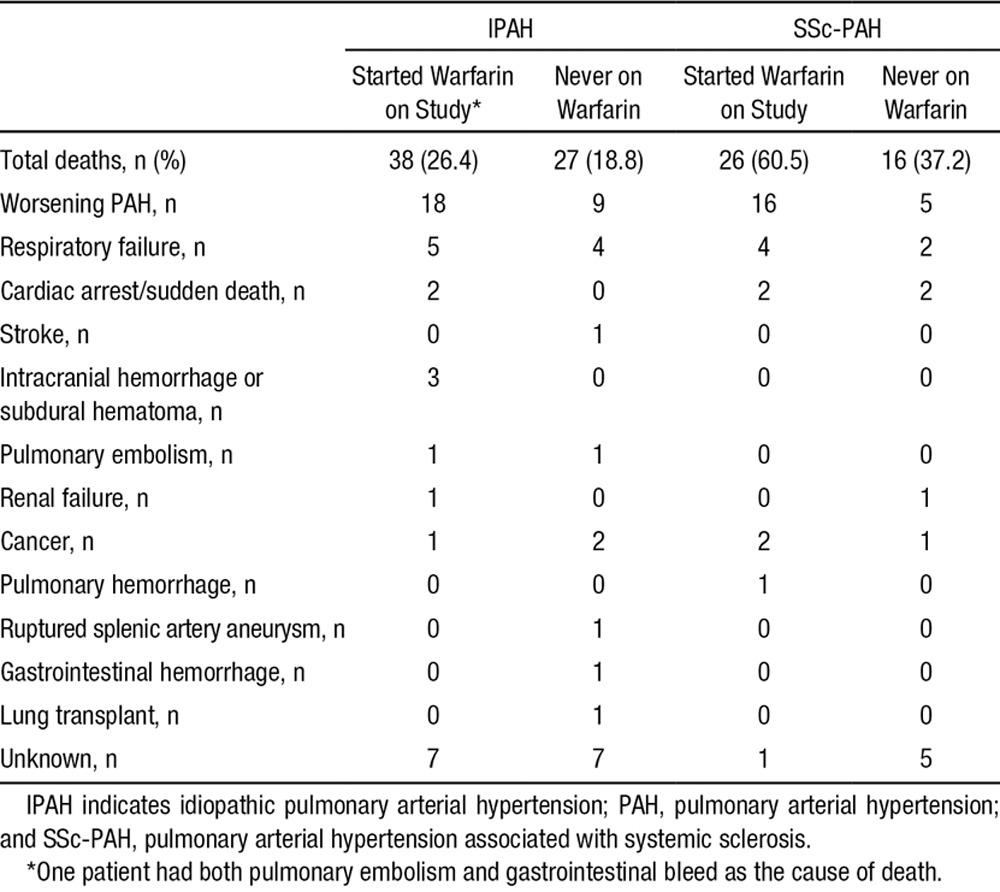
Reasons for Death

## Discussion

Our study shows no significant survival advantage in IPAH patients treated with warfarin in comparison with matched controls never treated with warfarin. This is further supported by the sensitivity analysis, which shows no survival advantage after adjustment for diagnosis status, PAH medication, and REVEAL Risk Score. Although SSc-PAH patients treated with warfarin had decreased survival in comparison with matched controls in the unadjusted analysis, SSc-PAH patients receiving warfarin were inherently sicker; no survival difference associated with warfarin therapy was present with adjustment for disease severity. After accounting for the high warfarin discontinuation rate in our study, the sensitivity analysis confirmed the observation of increased SSc-PAH patient mortality even after adjusting for disease severity at enrollment. Thus, analyses of the REVEAL Registry data provided no conclusive evidence to support the use of warfarin to reduce mortality in PAH patients. These data are in concordance with a Bayesian analysis of observational data from a longitudinal cohort of patients with SSc-PAH or IPAH, which demonstrated a low probability of a survival benefit with warfarin.^25^ In this Bayesian analysis, patients with SSc-PAH and IPAH who received warfarin were also determined to be sicker than patients not receiving warfarin, similar to COMPERA^15^ and to our REVEAL Registry population.^25^ In other disease states such as atrial fibrillation, warfarin has been shown to be effective in reducing the risk of morbid events (including stroke and systemic thromboembolism),^26^ with a recommended INR generally between 2.0 and 3.0.^26,27^ Importantly, the presumed goal of anticoagulant therapy in PAH is prevention of mortality, with a target INR of 1.5 to 2.5. The INR values for the patient population in the REVEAL Registry were within the American Heart Association recommended range for PAH^28^ and were below the levels of anticoagulation recommended for other conditions.^26,27^ Therefore, the risk of bleeding was likely minimized, because bleeding risk for patients treated with warfarin increases with increasing INR, particularly above a level of 4.^29^ Indeed, we did not detect an increased risk of death from bleeding, although morbid events were not specifically collected to detect adverse events related to warfarin use. Surprisingly, adherence to warfarin therapy in this cohort was poor in both cohorts. During the 3-year study, mean warfarin exposure was 1 year with a high discontinuation rate for both IPAH and SSc-PAH patients, suggesting a particularly poor tolerance to anticoagulation in PAH patients enrolled in the REVEAL Registry, a registry that represents current practice for treatment of PAH in the United States.

The current analysis is specifically designed to include new warfarin starts and excludes previously anticoagulated patients. This design is deliberate; to address the issue of immortal time bias,^22^ we focus on new warfarin starts and, hence, evaluate the possibility of anticoagulation-attributable survival. In contrast, the COMPERA analysis included patients who were anticoagulated (mostly with warfarin) at the time of enrollment, and thus did not account for immortal time bias. Importantly, COMPERA showed a positive correlation between anticoagulation and survival in IPAH patients only.^15^ Of note, both the REVEAL Registry and COMPERA revealed no positive association between anticoagulation and survival in SSc-PAH patients. This major difference in study design and immortal time bias might explain the discrepant results between the REVEAL Registry and the COMPERA-derived analyses in IPAH patients. The extent of this immortal time bias depends on the length of time occurring between diagnosis and initiation of warfarin (ie, immortal time) and whether this immortal time was misclassified or excluded. Immortal time refers to the period of time patients had to survive to initiate warfarin and be included in the warfarin cohort rather than the nonwarfarin cohort. For example, survival estimates may be biased in favor of the warfarin group if a lengthy period of prewarfarin survival is instead attributed to longer survival for warfarin patients (online-only Data Supplement Figure I). On the other hand, if patients typically initiate warfarin quickly after diagnosis, immortal time bias would be minimal and unlikely to fully explain the differences between the REVEAL Registry and COMPERA results.^15^ As such, immortal time bias could not definitively be excluded in COMPERA.

Other potential explanations for the discrepancy in results may include differences in patient characteristics between the 2 registries. The COMPERA cohort included more males than females and the patients were older. Moreover, the older age of patients in COMPERA is an important difference; patients 65 years or older had poorer survival despite having less severe (presumably advantageous) hemodynamic impairment.^30^ In addition, the use of prostacyclins in PAH care differs greatly between Germany, France, and the United States. In France and the United States, parenteral prostacyclins are initiated routinely**—**and early**—**as long-term therapy for patients presenting with NYHA/WHO functional class III/IV PAH. In contrast, in Germany, parental prostacyclin therapy is reserved mainly as a rescue therapy for hospitalized patients who are then discharged on oral therapies. Similarly, differences exist in PAH anticoagulation practices between the United States and Europe. In Europe, the target INR for PAH anticoagulation is 2.0 to 3.0^6^ and temporary interruptions are usually bridged with heparin or analogues. In contrast, the US PAH centers target an INR of 1.5 to 2.5 for PAH patients^28^ with no bridging for temporary interruptions. Of note, INR levels were not reported in COMPERA, but are reported in the REVEAL Registry.

Limitations of our study include the inherent limitations of observational registry-derived data and the retrospective nature of the report.^31^ Additionally, a high discontinuation rate of warfarin was observed, consistent with practice patterns in US PAH centers and suggestive of poor tolerance of anticoagulation in PAH patients. We attempted to account for this high rate of discontinuation by using a time-varying covariate model as a sensitivity analysis. Although both the matched and sensitivity analyses were adjusted for disease severity as captured by the REVEAL Risk Score, the anticoagulation arm was characterized by parameters consistent with disease severity. For example, a greater proportion of patients on anticoagulation received PAH parenteral and combination therapy, and had higher brain natriuretic peptide and NYHA/WHO functional class, which may well reflect that sicker PAH patients were more likely to be anticoagulated at baseline. The REVEAL Risk Score encompasses PAH risk factors including demographic data, comorbidities, NYHA/WHO group I subgroup, NYHA/WHO functional class, vital signs, 6-minute walk distance, and brain natriuretic peptide along with echocardiographic, pulmonary function testing, and hemodynamic risk factors, in a risk score prognostic model.^32^ Although it is evident that the REVEAL Risk Score captures most, if not all, known prognostic risk factors in PAH, the possibility of an unknown possible factor cannot be excluded. Furthermore, the REVEAL Risk Score has been validated in both incident and prevalent patients and has been cross-validated using the French registry database.^33^ Although a predictive score combining right ventricular function and right atrial function indices for predicting outcome has been validated in PAH patients, this score was not shown to be statistically different from the REVEAL Risk Score.^34^ Because no other general risk scores have been evaluated or validated in PAH, the use of the REVEAL Risk Score is currently the best tool that accounts for differences in disease severity.

Our study is novel in that it consists of a rigorous analysis of a large database with inclusion of new starts on warfarin, exclusion of immortal time bias, as well as adjustment for disease severity as measured by the REVEAL PAH-specific Risk Scores. This study also reports on the length of anticoagulant therapy and on the level of anticoagulation measured by INR during warfarin use, all of which contribute to the strength and accuracy of the results. In addition, statistical modeling was done to address the limitations as well as to validate the matching analysis, by performing a time-varying covariate model as an analogous sensitivity analysis.

In conclusion, in IPAH patients in the REVEAL Registry, there is insufficient evidence to conclude that the initiation of warfarin is associated with improved survival. Furthermore, similar results were observed in SSc-PAH patients. Our findings differ from COMPERA in regard to IPAH patients but are concordant with COMPERA and other studies with regard to the use of anticoagulation in SSc-PAH patients. Further studies are required to resolve this ongoing controversy.

## Acknowledgments

Medical writing and editorial assistance was provided by Terri Schochet, PhD, of AlphaBioCom, LLC, King of Prussia, PA. The authors thank Simona Neumann, PhD, of Actelion Pharmaceuticals US, Inc., for final draft-writing support.

## Sources of Funding

Actelion Pharmaceuticals US, Inc., is the sponsor of the REVEAL Registry and provided funding and support for the analysis presented.

## Disclosures

Dr Preston has served as a consultant for Actelion Pharmaceuticals, Bayer, Gilead, and United Therapeutics; and has received research support from Actelion Pharmaceuticals, AIRES, Bayer, Gilead, and United Therapeutics. Dr Roberts has no conflicts of interest to report. At the time the research was conducted, D. Miller was an employee of ICON Clinical Research the biostatistics CRO for the REVEAL Registry. G. Sen is an employee of ICON Clinical Research the biostatistics CRO for the REVEAL Registry. Dr Selej holds stock or stock options in and is an employee of Actelion Pharmaceuticals. At the time of writing, W. Benton held stock options in and was an employee of Actelion Pharmaceuticals. Dr Hill has served as a consultant for Actelion Pharmaceuticals, Bayer, and Gilead; has received research grants from Actelion Pharmaceuticals, Bayer, Gilead, Ikaria, Reata, and United Therapeutics; and serves on the Data and Safety Monitoring Board for the BEAT study sponsored by Lung LLC. Dr Farber has received research grants from Gilead and United Therapeutics; consulting fees from Gilead, Actelion Pharmaceuticals, United Therapeutics, Bayer, and Ikaria; and has served on a speaker’s bureau or given presentations on behalf of Actelion Pharmaceuticals, Gilead, and Bayer.

## Supplementary Material

## CLINICAL PERSPECTIVE

Long-term warfarin anticoagulation is recommended for patients with idiopathic pulmonary arterial hypertension and in patients with pulmonary arterial hypertension associated with systemic sclerosis (SSc-PAH). In this analysis of data from the Registry to Evaluate Early and Long-term PAH Disease Management (REVEAL) Registry, we found that treatment with warfarin conferred no survival benefit in patients with idiopathic pulmonary arterial hypertension or with SSc-PAH in comparison with matched controls. Importantly, SSc-PAH patients receiving warfarin had increased mortality in comparison with warfarin-naïve patients. Combined with a recent analysis of idiopathic pulmonary arterial hypertension and SSc-PAH patients exposed to warfarin from the European registry Comparative, Prospective Registry of Newly Initiated Therapies for Pulmonary Hypertension (COMPERA), our observations suggest that warfarin therapy should not be recommended or instituted in patients with SSc-PAH. However, in patients with idiopathic pulmonary arterial hypertension, a definitive recommendation is not as clear, because, in contrast to our findings, a survival benefit was observed in these patients from the COMPERA registry. As such, until there is a robust trial prospectively examining this cohort—which is unlikely—clinical judgment and the risk of untoward events should be the critical factors underlying the decision to initiate warfarin therapy.
