# Supplementary material for: Effect of Warfarin Treatment on Survival of Patients With Pulmonary Arterial Hypertension (PAH) in the Registry to Evaluate Early and Long-Term PAH Disease Management (REVEAL)
Source: Circulation. 2015 Dec 21;132(25):2403–11. doi: 10.1161/CIRCULATIONAHA.115.018435 (PMC4689180; doi:10.1161/CIRCULATIONAHA.115.018435)

SUPPLEMENTAL MATERIAL

Supplemental Table 1. Time Varying Warfarin Analysis Among Warfarin-naïve Patients at Enrollment: Adjusted Cox Proportional Hazard Model

	**IPAH**<br>**N = 655**<br>Started Warfarin on Study, n = 163<br>No on-study warfarin use, n = 492			**SSc-PAH**<br>**N = 342**<br>Started Warfarin on Study, n = 55<br>No on-study warfarin use, n = 287		
	**Warfarin Hazard Ratio**	**95% CI**	***P* value**	**Warfarin Hazard Ratio**	**95% CI**	***P* value**
**Adjusted*† : past use**	0.84	0.59–1.20	0.34	1.49	1.01–2.20	0.046
**Adjusted*‡ : current use**	0.96	0.66–1.39	0.82	1.57	1.04–2.36	0.031

Note: blinded trial patients were excluded at enrollment.
* Adjusted for diagnosis status, ERA/PDE5/PGI2 at enrollment, and risk score at enrollment

† Any postbaseline on-study warfarin use.
‡ Any postbaseline on-study warfarin use within the previous year.

CI, confidence interval; ERA, endothelin receptor antagonist; IPAH, idiopathic PAH; PDE5, phosphodiesterase type 5 inhibitor; PGI2, prostacyclin; SSc-PAH, pulmonary arterial hypertension associated with systemic sclerosis.

Supplemental Figure 1: Demonstration of immortal time bias

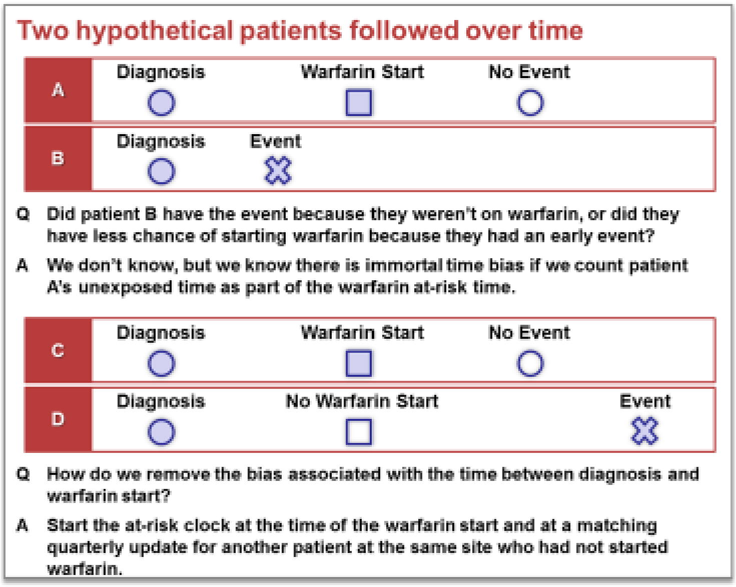

Supplement: Supplementary file 1 [file cir-132-2403-s001.pdf]
